# MicroRNA-99 Family Targets AKT/mTOR Signaling Pathway in Dermal Wound Healing

**DOI:** 10.1371/journal.pone.0064434

**Published:** 2013-05-28

**Authors:** Yi Jin, Stéphanie D. Tymen, Dan Chen, Zong Juan Fang, Yan Zhao, Dragan Dragas, Yang Dai, Phillip T. Marucha, Xiaofeng Zhou

**Affiliations:** 1 Center for Molecular Biology of Oral Diseases, College of Dentistry, University of Illinois at Chicago, Chicago, Illinois, United States of America; 2 Center for Wound Healing and Tissue Regeneration, College of Dentistry, University of Illinois at Chicago, Chicago, Illinois, United States of America; 3 Department of Oral and Maxillofacial Surgery, the First Affiliated Hospital, Sun Yat-Sen University, Guangzhou, China; 4 Department of Bioengineering, College of Engineering, University of Illinois at Chicago, Chicago, Illinois, United States of America; 5 UIC Cancer Center, University of Illinois at Chicago, Chicago, Illinois, United States of America; 6 Department of Periodontics, College of Dentistry, University of Illinois at Chicago, Chicago, Illinois, United States of America; University of Nevada School of Medicine, United States of America

## Abstract

Recent studies suggest that microRNAs play important roles in dermal wound healing and microRNA deregulation has been linked with impaired wound repair. Here, using a mouse experimental wound healing model, we identified a panel of 63 differentially expressed microRNAs during dermal wound healing, including members of miR-99 family (miR-99a, miR-99b, miR-100). We further demonstrated that miR-99 family members regulate cell proliferation, cell migration, and AKT/mTOR signaling. Combined experimental and bioinformatics analyses revealed that miR-99 family members regulate AKT/mTOR signaling by targeting multiple genes, including known target genes (e.g., IGF1R, mTOR) and a new target (AKT1). The effects of miR-99 family members on the expression of IGF1R, mTOR and AKT1 were validated at both the mRNA and protein levels. Two adjacent miR-99 family targeting sites were identified in the 3′-UTR of the AKT1 mRNA. The direct interaction of miR-100 with these targeting sites was confirmed using luciferase reporter assays. The microRNA-100-directed recruitment of AKT1 mRNA to the RNAi-induced silencing complex (RISC) was confirmed by a ribonucleoprotein-IP assay. In summary, we identified a panel of differentially expressed microRNAs which may play important roles in wound healing. We provide evidence that miR-99 family members contribute to wound healing by regulating the AKT/mTOR signaling.

## Introduction

The wound healing process is composed of a complex series of molecular events triggered by an injury to prevent infection, repair the damaged tissue and restore its function. Such intricate physiological processes are well-orchestrated by a variety of known and unknown factors to ensure a successful outcome. Indeed, impaired and aberrant wound healing is a significant clinical problem which affects approximately 6.5 million people in the United States. Improvement in managing impaired wound healing requires better understanding of the wound healing process at the molecular level. While many attempts have been made to investigate molecular events in wound healing, most efforts have been focused on protein coding genes. Our knowledge of non-coding genes (e.g., microRNA) and their contributions to wound healing is relatively limited. MicroRNAs are a class of small non-coding RNAs of approximately 22 nucleotides in length that are endogenously expressed in mammalian cells. They regulate the expression of their target genes mainly at the post-transcriptional level by repressing translation and/or facilitating mRNA degradation. MicroRNAs have been shown to regulate many developmental and physiological processes, as well as a number of disease processes. Recent findings report their involvement in psoriasis [1], in autoimmune diseases affecting the skin, such as systemic lupus erythematosus [2], in systemic sclerosis [3], in hair follicle morphogenesis (ablation of microRNAs from keratinocytes causes several defects, such as evagination instead of invagination) [4], and in skin carcinogenesis [5], [6]. Roles for microRNA have also been proposed in wound healing [7]–[9], and microRNA deregulation has been linked with impaired wound healing [10]–[14].

In this study, we identified a panel of differentially expressed microRNAs during dermal wound healing, including members of miR-99 family. We further demonstrated that the miR-99 family regulates the wound healing process by targeting multiple genes in the AKT/mTOR signaling pathway, which plays a major role in cell migration and proliferation that contribute to the replenishment of lost tissues after injury.

## Materials and Methods

### Animals and Experimental Wound Model

The animal study was approved by the Institutional Animal Care and Use Committee at University of Illinois at Chicago. Eight-week old female SKH-1e mice were obtained from Charles River, Inc. (Wilmington, MA). Mice were housed in conventional cages, five animals per cage, under a 12∶12 light:dark cycle (starting at 18∶00), before and throughout the experiments. Animals were allowed 1–2 weeks to acclimate to the cages before the start of the experiment.

Experimental wounds were created as previously described [15]. In brief, mice were anesthetized with 250 µL doses of ketamine-xylazine-saline solution (ratio 4∶1:35) consisting of ketamine 100 mg/Kg and xylazine 5 mg/Kg, administered intra-peritoneally. The dorsal skin was cleaned with isopropanol pads and two full-thickness wounds were created below the shoulder blades (one on each side of the midline) using a sterile 3.5 mm biopsy punch (Miltex Inc., York, PA). Mice were anesthetized and the wounds were harvested one and five days post-wounding with a 6 mm biopsy punch (Miltex Inc., York, PA) before the mice were euthanized. The tissue samples from the same animal were pooled and stored in RNAlater (Sigma-Aldrich) immediately after harvesting.

### RNA Isolation and Microarray Analysis

Tissue samples were homogenized in TRIzol (Invitrogen) using a Tissue-Tearor (Cole-Parmer); RNA was isolated by standard protocol. In brief, total RNA was extracted by chloroform extraction, isopropanol precipitation, 75% ethanol wash and resuspension in DEPC-treated water, and then quantified by spectrophotometry. The microRNA microarray analysis was performed by LC Sciences, LLC (Houston, TX) using their MRA-1002 miRmouse v15 microarrays. One-way ANOVA test was used to compute the p value. Clustering and principal component analysis (PCA) were performing using Cluster 3.0 and TreeView [16], [17]. MicroRNA target prediction and pathway analysis was performed using DIANA-mirPath [18]. The microarray dataset has been submitted to ArrayExpress Archive (accession number: E-MTAB-1518).

### Cell Culture and Transfection

The human immortal keratinocyte cell line (HaCaT [19]) was maintained in high glucose DMEM medium (Gibco) supplemented with 10% FBS, 100 units/ml penicillin, and 100 µg/ml streptomycin (Invitrogen) at 37°C in a humidified incubator containing 5% CO_2_. For functional analysis, miR-99a, miR-99b, miR-100 or control microRNA mimic (Dharmacon) was transfected into the cells using DharmaFECT Transfection Reagent 1 as described previously [20], [21]. To test the effect of PI3K/AKT and mTOR signaling pathways, cells were treated with 50 uM LY294002 (PI3 Kinase inhibitor) or 10 nM Rapamycin (mTOR inhibitor). To test the effect of IGF1 and serum stimulation, cells were starved in serum-free DMEM medium overnight, and then incubated with 100 ng/ml IGF1 or 20% serum for 30 minutes.

### Cell Proliferation, Migration and Apoptosis Assays

Cell proliferation was measured using the MTT [3-(4,5-dimethylthiazol-2-yl)-2,5-diphenyl-2H-tetrazolium bromide] assay as described previously [22]. In brief, at 48 h post-transfection, the transfection medium in each well was replaced by 100 µl of fresh serum-free medium with 0.5 g/l MTT. After incubation at 37°C for 4 h, the MTT medium was removed by aspiration and 50 µl of DMSO was added to each well. After incubation at 37°C for a further 10 min, absorbance at 540 nm for each sample was measured using a plate reader. In addition, cell proliferation was also measured using a CyQUANT Direct Cell Proliferation Assay kit (Invitrogen/Molecular Probes) according to manufacture’s instruction, and quantified with a fluorescence plate reader with excitation at 485 nm and emission detection at 530 nm.

Cell migration was measured using a scratch assay as described previously [21]. In brief, cells were seeded in 12-well plates and cultured to confluence. Wounds of 1 mm width were created with a plastic scraper, and cells were washed and incubated in a serum-free medium. 24 hours after wounding, cultures were fixed and observed under a microscope. A minimum of 5 randomly chosen areas were measured. In addition, cell migration was also measured using a trans-well assay as described previously [23] using BD BioCoat Control Cell Culture Inserts (containing an 8.0 *µ*m PET Membrane without matrix).

The apoptosis was measured using the Annexin V-FITC Apoptosis Detection Kit (Invitrogen) and measured with a flow cytometer (FACScalibur, Becton–Dickinson) as previously described [22].

### Western Blot Analysis

Western blots were performed as described previously [21] using antibodies specific for IGF1R, mTOR, AKT1/2/3, p70S6K, phospho-p70S6K(Thr389), 4E-BP1, phospho-4E-BP1(Thr37/Thr46) (Cell Signaling), and β-actin (Sigma-Aldrich), and an immuno-star HRP substrate Kit (Bio-RAD).

### Quantitative RT-PCR Analysis

The relative expression levels of miR-99a, miR-99b and miR-100 were determined by TaqMan microRNA assays as previously described [24]. The relative mRNA levels of IGF-1R, mTOR, AKT1 and FOSL1 were determined by quantitative two-step RT-PCR assay with gene specific primer sets (Origene) as described before [25]. The relative microRNA and mRNA levels were computed using the 2^−delta delta Ct^ analysis method, where U6 and β-actin were used as internal controls, respectively.

### Dual-Luciferase Reporter Assay

The luciferase reporter gene construct containing 2 adjacent miR-99 family targeting sites from the 3′-UTR of AKT1 mRNA was created by cloning an 81-bp fragment into the XbaI site on the 3′-UTR of the luciferase gene in the pGL3-Control firefly luciferase reporter vector (Promega) as described previously [26]. The corresponding mutant constructs were created by replacing the seed regions (positions 2–8) of the miR-99 family binding sites with 5′-TTTTTTT-3′. All constructs were verified by sequencing. The reporter constructs and the pRL-TK vector (Promega) were co-transfected using Lipofectamine 2000 (Invitrogen). The luciferase activities were then determined as described previously [20] using a GloMax 20/20 luminometer (Promega). Experiments were performed in quadruplicate.

### Ribonucleoprotein-IP (RIP-IP) Assay

RIP-IP assays were performed as described previously [27]. Briefly, cells were co-transfected with a pIRESneo-FLAG/HA-Ago2 expression vector (Addgene plasmid 10822, Addgene Inc.) and miR-100 mimic, miR-138 mimic or non-targeting microRNA mimic (Dharmacon). 48 h after transfection, cells were washed and lysed in radioimmune precipitation buffer (Sigma) containing 10% proteinase inhibitor cocktail (Sigma), 1 mM PMSF (Fluka), and 100 units/ml SUPERase·In (Ambion). The samples were then subjected to centrifugation for 30 min at 14,000 rpm, and the supernatants were collected. A fraction of the whole cell lysate was saved for RNA isolation, and the remaining lysate was subjected to immunoprecipitation (IP) using anti-FLAG M2 affinity gel (Sigma). RNA from the whole cell lysate and the RIP-IP fraction was extracted with QIAzol and purified by miRNeasy mini kit (Qiagen). The relative mRNA levels of the AKT1, IGF1R, mTOR, and FOSL1 were determined using a quantitative two-step RT-PCR as described. The relative enrichment of mRNA in the RIP-IP fractions was computed based on the ratio of relative mRNA levels in the RIP-IP fractions and the relative mRNA levels in the whole cell lysates as described previously [27].

## Results and Discussion

**Figure 1:**
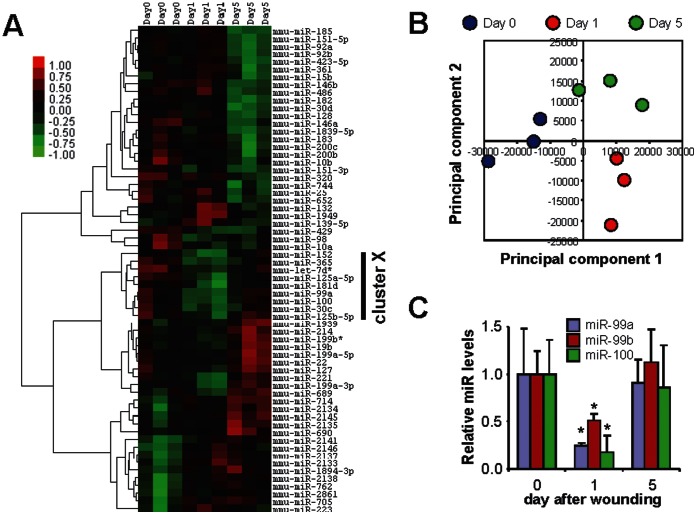
Differential expression of microRNA during dermal wound healing. **A**) MicroRNA microarray profiling analysis was performed on skin biopsies from unwounded mice, and from mice 1 day or 5 days post-wounding (3 mice per groups) as described in Material and Methods section. The levels of 63 microRNAs were changed (p<0.05). Hierarchical clustering analysis was performed based on the differentially expressed microRNAs. A 9-microRNA cluster (mmu-miR-152, mmu-miR-365, mmu-let-7d*, mmu-miR-125a-5p, mmu-miR-181d, mmu-miR-99a, mmu-miR-100, mmu-miR-30c, mmu-miR-125b-5p, which exhibited statistical significant down-regulation on day 1 and returned to basal level on day 5) was named as cluster X (marked by solid bar on the right). **B**) Principle Component (PC) analysis was performed based on the differentially expressed microRNAs. The first 2 PCs were plotted. Blue: unwounded skin (day 0), Red: 1 day post-wounding, Green: 5 days post-wounding. **C**) The differential expression of miR-99a, miR99b and miR-100 were confirmed by quantitative RT-PCR in additional mice at 0, 1 and 5 days post-wounding (6 mice per groups). *: p<0.05.

Using a well-established experimental mouse dermal wound healing model [15], we performed microRNA expression profiling analysis on skin samples of unwounded mice, and skin biopsy samples harvested at 1 and 5 days post-wounding. As shown in **Figure 1A** and **Table S1**, the levels of 63 microRNAs were changed during wound healing. Hierarchical clustering analysis revealed several groups of microRNAs that exhibit similar expression patterns, including a 9-microRNA group (mmu-miR-152, mmu-miR-365, mmu-let-7d*, mmu-miR-125a-5p, mmu-miR-181d, mmu-miR-99a, mmu-miR-100, mmu-miR-30c, mmu-miR-125b-5p, named as cluster X in **Figure 1A**) which was down-regulated during the early phase of wound healing (day 1) as compared to unwounded skin (day 0), and returned to basal level during the later phase of wound healing (day 5) (**Table 1**). Principal Component Analysis (PCA) was performed based on the differentially expressed microRNAs, and apparent separations among groups were observed in relation to the phase of wound healing (unwounded, early phase, and later phase of wound healing) (**Figure 1B**).

**Table 1:** Differential expression of microRNA in would healing^a^.

microRNA	Day 0	Day 1	Day 5	p-value
mmu-miR-152	1.000	0.538	1.035	0.0015
mmu-miR-365	1.000	0.139	0.437	0.003
mmu-miR-125a-5p	1.000	0.317	0.588	0.0044
mmu-miR-99a	1.000	0.242	0.912	0.0118
mmu-let-7d*	1.000	0.458	0.694	0.0152
mmu-miR-100	1.000	0.180	0.859	0.0154
mmu-miR-181d	1.000	0.300	0.643	0.0272
mmu-miR-30c	1.000	0.544	0.846	0.041
mmu-miR-125b-5p	1.000	0.544	0.980	0.0425

a For each time point (day 0, 1, 5 post-wounding), mean relative microRNA levels were computed based on 3 independent microarray experiments on 3 animals. A panel of 9 microRNAs (cluster X from **Figure 1A**) that were down-regulated during the early phase of wound healing (day 1) and returned to basal level during the later phase of wound healing (day 5) are presented. The complete list of differentially expressed microRNAs is presented in **Table S1**.

To understand the role(s) of microRNA in complex biological processes (i.e., wound healing), it is important to define relevant molecular pathways that are regulated by microRNA and their target genes. Bioinformatics analysis (target prediction and pathway analysis) revealed that a number of biological pathways are targeted by this panel of 63 differentially-expressed microRNAs (**Table S2**), and the 9-microRNA cluster X regulates a subset of these pathways, including mTOR and MAPK signaling pathways (**Table 2** and **Table S3**). Among the cluster X microRNAs, mmu-miR-99a and mmu-miR-100 are members of miR-99 family. The miR-99 family is one of the evolutionarily most ancient microRNA families whose origin dates back before the bilaterian ancestor [28], [29], and the sequences of the mature microRNAs are identical in human and mouse. Recent studies suggest that the miR-99 family regulates mTOR signaling in cancer cells [30]–[32]. As shown in **Figure 1C**, differential expression of mmu-miR-99a and mmu-miR-100 during wound healing was confirmed by quantitative RT-PCR in additional animals (n = 10). Mmu-miR-99b (the 3rd member of the miR-99 family) is also differentially expressed during dermal wound healing (p<0.05) in mice. Down-regulation of hsa-miR-99b and hsa-miR-100 was confirmed in human skin wounds (**Figure S1**), while no apparent difference was observed in the expression of hsa-miR-99a.

**Table 2:** Common molecular pathways regulated by differentially expressed microRNAs^a^.

KEGG Pathway	Pathway ID	# of genes targeted in the pathway (Union)	−ln(p-value)^b^
Long-term potentiation	mmu04720	24	21.62
mTOR signaling pathway	mmu04150	19	16.35
MAPK signaling pathway	mmu04010	56	15.76
Axon guidance	mmu04360	34	15.71
Glioma	mmu05214	21	15.7
Ubiquitin mediated proteolysis	mmu04120	34	15.15

a 9 differentially expressed microRNAs (in cluster X from **Figure 1A**, including miR-152, miR-365, let-7d*, miR-125a-5p, miR-181d, miR-99a, miR-100, miR-30c, miR-125b-5p) were used for the analysis. Pathway analysis with complete list of 63 differentially expressed microRNAs is presented in **Table S2**.

b Computed using DIANA-mirPath [18]. Cut-off −ln(p-value) of 15 was used. Complete list of pathways regulated by 9 differentially expressed microRNAs (in cluster X from **Figure 1A**) is presented in **Table S3**.

**Figure 2:**
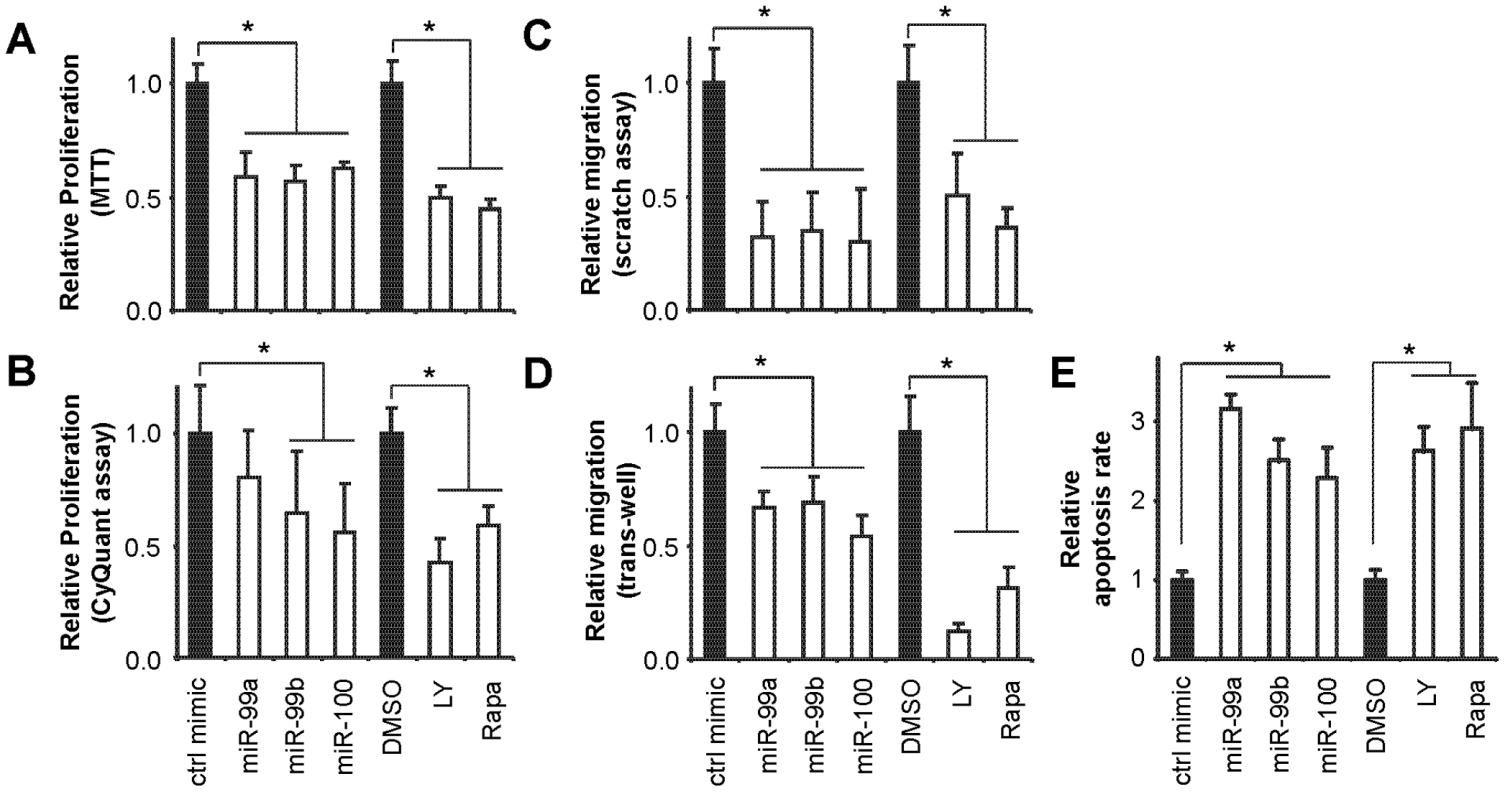
The effect of the miR-99 family on cell proliferation and cell migration in skin keratinocytes. HaCaT cells were transfected with mimics for miR-99a, miR-99b, miR-100 or negative control mimic, or treated with PI3 Kinase Inhibitor LY294002 (LY), mTOR Inhibitor Rapamycin (Rapa) or vehicle alone (DMSO). Cell proliferation was measured by MTT assay (**A**) and CyQUANT assay (**B**). Cell migration was measured by scratch assay (**C**) and trans-well assay (**D**). Apoptosis was measured by flow cytometry (**E**). The experiments were performed in quadruplicates. *: p<0.05.

The miR-99 family members (miR-99a/b and miR-100) have been shown to regulate cell proliferation and cell migration in several types of cancer of epithelial origin [24], [33], [34], however, their role(s) in wound healing are not well defined. As such, we chose to further explore the functional role of the miR-99 family in our study. It is well-established that proliferation and migration of keratinocytes from the wound margin result in re-epithelialization during wound healing [35]. As shown in **Figure 2A**, ectopic transfection of miR-99a, miR-99b, and miR-100 mimic to HaCaT cells led to a statistically significant down-regulation in cell proliferation (measured by MTT assay). Statistically significant down-regulation in cell proliferation was also observed in cells treated with miR-99b and miR-100 mimic as measured by quantifying the DNA content (CyQUANT assay, **Figure 2B**). An apparent reduction in cell proliferation was also observed in cells treated with miR-99a mimic, however, the difference was not statistically significant. As shown in **Figure 2C and 2D**, ectopic transfection of miR-99a, miR-99b, and miR-100 mimic to HaCaT cells led to a statistically significant down-regulation in cell migration (measured by scratch assay and trans-well assay) as compared to the cells treated with control mimic. As shown in **Figure 2E**, ectopic transfection of miR-99a, miR-99b, and miR-100 mimic led to a statistically significant increase in apoptosis as compared to control mimic treatment. When cells were treated with LY294002 (PI3-kinase inhibitor) or Rapamycin (mTOR inhibitor), statistically significant reductions were observed in relative cell proliferation (**Figure 2A and 2B**) and cell migration (**Figure 2C and 2D**), and statistically significant increase in apoptosis was observed (**Figure 2E**). These results are consistent with our bioinformatics analysis and the recent observations based on cancer cells [30]–[32] suggesting that the members of miR-99 family regulate cell proliferation, apoptosis and migration by targeting the mTOR signaling pathway, as well as the PI3K/AKT pathway which is upstream of mTOR. As shown in **Figure S2**, when cells were treated with anti-miR-100 LNA, statistically significant increases in cell proliferation and migration were observed.

**Figure 3:**
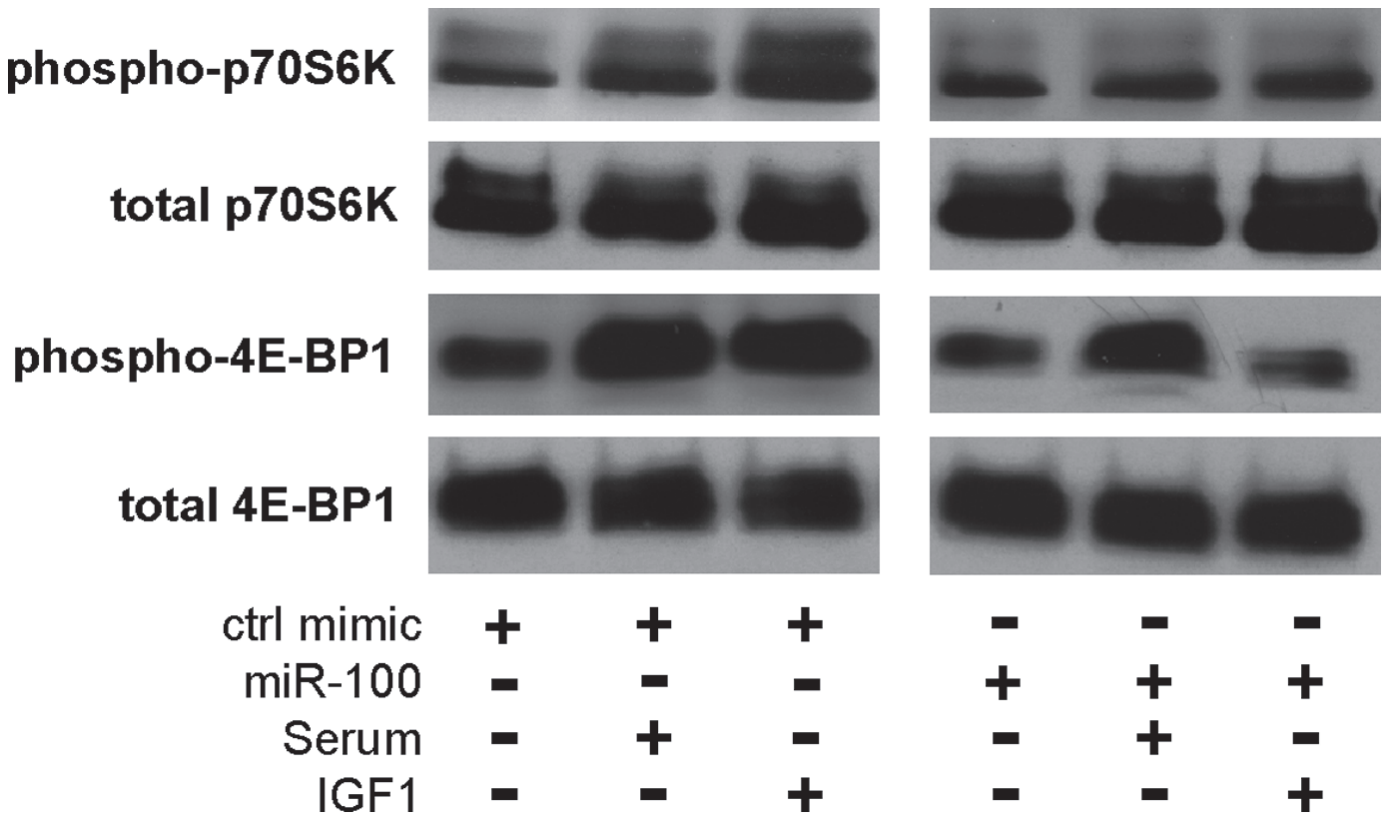
The effect of miR-100 on the phosphorylation of p70S6K and 4E-BP1. HaCaT cells were transfected with either negative control mimic (**A**) or miR-100 mimic (**B**), and then stimulated with IGF1 or serum. Western blot analysis was performed to assess the levels of total p70S6K protein, phosphorylated p70S6K, total 4E-BP1 protein, and phosphorylated 4E-BP1.

To directly demonstrate the effect of miR-99 family on PI3K/AKT and mTOR signaling, we investigated the effect of miR-100 on the phosphorylation of p70 S6 Kinase (p70S6K) and eukaryotic translation initiation factor 4E binding protein 1 (4E-BP1), two important signaling molecules that lie downstream of PI3K/AKT and mTOR [36]. The activities of both signaling molecules are controlled by multiple phosphorylation events, including phosphorylation of Thr389 on p70S6K, and phosphorylation of Thr37 and Thr46 on 4E-BP1. As shown in **Figure 3**, serum- and IGF1-treatment induced phosphorylation of p70S6K and 4E-BP1 in HaCaT cells pre-treated with control mimic, while the total p70S6K and total 4E-BP1 remained relatively constant in these cells. However, when cells were pre-treated with miR-100 mimic, the IGF1-induced phosphorylation of p70S6K and 4E-BP1 were reduced dramatically. Apparent reduction in the serum-induced phosphorylation of p70S6K and 4E-BP1 was also observed in cells pre-treated with miR-100. As expected, both LY294002 and Rapamycin treatments reduced the phosphorylation of p70S6K and 4E-BP1 (data not shown). These results provide further evidence supporting a role for the miR-99 family in regulating PI3K/AKT and mTOR signaling.

**Figure 4:**
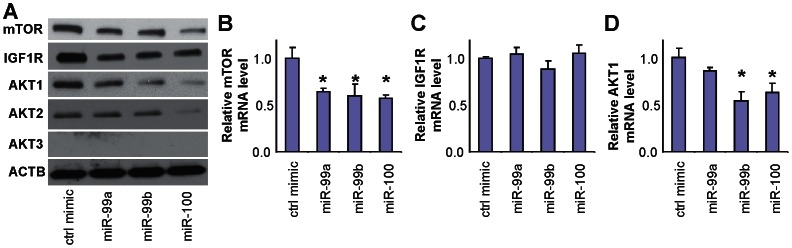
The effect of the miR-99 family on the expression of mTOR, IGF1R and AKT. HaCaT cells were transfected with mimics for miR-99a, miR-99b, miR-100 or negative control mimic. Western blot analysis was performed to measure the protein levels of mTOR, IGF1R, AKT1, AKT2 and AKT3 (**A**). Quantitative RT-PCR assays were performed to measure the mRNA levels of mTOR (**B**), IGF1R (**C**), and AKT1 (**D**). *: p<0.05.

IGF1R and mTOR, two major players in PI3K/AKT and mTOR signaling pathways, have previously been suggested as direct targets of the miR-99 family in different types of cancer cells [24], [30]–[32]. Activation of IGF1R and mTOR has also been associated with re-epithelialization of dermal wounds [37], [38]. Here, we confirmed the effects of the miR-99 family on the expression of IGF1R and mTOR in skin keratinocytes (HaCaT). As shown in **Figure 4A**, decreases in IGF1R and mTOR protein levels were observed in cells that were treated with miR-100, miR-99a or miR-99b. Significant reduction in mTOR mRNA level was observed in cells that were treated with miR-99a, miR-99b or miR-100 (**Figure 4B**). Interestingly, no statistically significant change in IGF1R mRNA level was observed in cells that were treated with miR-99a, miR-99b or miR-100 as measured by quantitative RT-PCR (**Figure 4C**). This is consistent with previous observations made in HaCaT cells [39], and suggests that the miR-99 family regulates IGF1R gene expression primarily by inhibiting translation in HaCaT cells. It is worth noting that we recently showed that both IGF1R protein and mRNA were significantly reduced in 2 head and neck cancer cell lines (UM1 and 1386Ln) when they were treated with mimics of miR-99 family members [24]. These results suggest that the same microRNA may utilize different mechanisms to down-regulate a gene in different cell types. More studies will be needed to define this interesting phenomenon.

In addition to IGF1R and mTOR, we also tested whether the miR-99 family regulates the expression of the AKT gene family. As shown in **Figure 4A**, the AKT1 protein level decreased in cells that were treated with miR-100, miR-99a or miR-99b. Decrease in the AKT2 protein level was also observed in cells treated with miR-100, but not in cells treated with miR-99a or miR-99b. AKT3 was not detectable in HaCaT cells. Reduction in the AKT1 mRNA level was also observed in cells treated with miR-99b or miR-100 (**Figure 4D**). An apparent decrease in AKT1 mRNA was also observed in cells treated with miR-99a, but the difference was not statistically significant. These miR-99 family-induced changes in IGF1R, mTOR and AKT1 expression were further validated in mouse wounds by directly applying the microRNA mimics to the dermal wounds. As shown in **Figure S3**, decreases in mTOR and AKT1 mRNA were observed in mouse dermal wounds that were treated with miR-100 mimic as compared to the wounds treated with control mimic. However, the differences were not statistically significant. The miR-100-mediated change in IGF1R mRNA level was less drastic. Taken together, these results suggested that the miR-99 family regulates PI3K/AKT and mTOR signaling pathways by targeting a number of key genes, including known target genes (IGF1R, mTOR), and the new target gene AKT1. We are in the process of optimizing the microRNA delivery strategy in our mouse experimental wound model, which will facilitate the further evaluation of the miR-99 effect on wound healing in the *in vivo* model. The outcome from these studies will provide a strong rationale and scientific foundation for developing novel microRNA-based therapeutic approaches for treating impaired wound healing.

**Figure 5:**
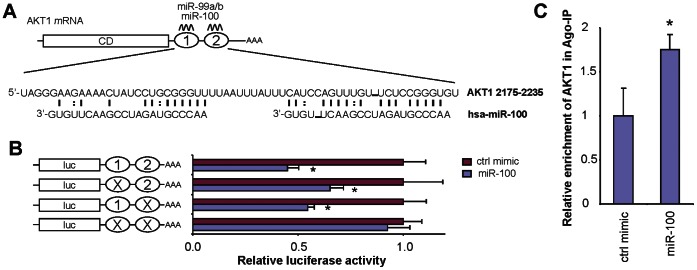
MicroRNA-100 directly interacts with AKT1 mRNA. **A**) Two adjacent targeting sites for miR-99a/b/100 were identified in AKT1 3′-UTRs (nt 2175 to nt 2235, NM_005163). **B**) Dual luciferase reporter assays were performed to test the interactions of miR-100 and the targeting sequences in the AKT1 mRNA using constructs containing the predicted targeting sequences and the corresponding mutants cloned into the 3′-UTR of the reporter gene. **C**) RIP-IP assays were performed to co-IP the Ago2 complexes from cells transfected with either miR-100 mimic or negative control mimic. qRT-PCR assays were performed on RNA samples isolated from the Ago2 co-IP fractions to measure the relative enrichment of the AKT1 mRNA. Data represent at least 3 independent experiments with similar results. *: p<0.05.

While the targeting sequences for miR-99 members on IGF1R and mTOR mRNAs have been previously identified and experimentally confirmed using luciferase reporter assays [24], [30]–[32], the direct interaction of miR-99 family and AKT1 mRNA has not been defined. Bioinformatics analysis revealed 2 adjacent miR-99 family targeting sites in the 3′-UTR of the AKT1 mRNA (**Figure 5A**). The AKT1 gene has 3 transcript variants, resulting from alternative utilization of 5′ exons. All 3 variants have identical 3′-UTR, and as such, they contain the same set of miR-99 family targeting sites. The sequence conservation among species, the predicted binding, and the calculated binding affinity (minimum free energy) of these sites were presented in **Figure S4**. No targeting site for the miR-99 family was indentified in the AKT2 mRNA sequence. To test whether the miR-99 family directly interacts with these predicted targeting sites in AKT1 mRNA, dual luciferase reporter assays were performed using constructs containing these targeting sites (**Figure 5B**). When cells were transfected with miR-100, the luciferase activities of the construct containing both targeting sites was significantly reduced as compared to the cells transfected with negative control. When the seed region of one of the two targeting sites was mutated, the miR-100-mediated reduction in luciferase activity was still observed. When both targeting sites were mutated, the effect of miR-100 on the luciferase activity was abolished. These results confirmed that miR-100 directly interacts with these targeting sites in AKT1 mRNA.

It is worth noting that while the expression of the AKT2 gene was also down-regulated by miR-100 treatment (**Figure 4A**), no targeting sequence was identified in AKT2 mRNA sequence. It is possible that miR-100 indirectly regulates AKT2 by targeting factors that control AKT2 gene expression. Alternatively, AKT2 may be regulated by miR-100 through a noncanonical targeting sequence. Additional studies are needed to explore the mechanisms that contribute to miR-100-mediated AKT2 expressional change.

Mature microRNAs form stable complexes with Argonaute proteins (such as Ago2), the core of the RNAi-induced silencing complex (RISC). The microRNA then directs RISC to bind to the mRNA molecules containing specific targeting sequences, and results in translational repression and/or enhanced mRNA degradation. To further confirm that miR-100 directly interacts with AKT1 mRNA, we tested the miR-100-mediated binding of RISC to AKT1 mRNA using an Ago2-based ribonucleoprotein-IP assay (RIP-IP). As shown in **Figure 5C**, the Ago2 co-IP fractions from cells treated with miR-100 mimic were significantly enriched in AKT1 mRNA as compared to cells treated with control mimic. As shown in **Figure S5A**, an apparent enrichment of IGF1R mRNA was also observed, however, the difference was not statistically significant. Interestingly, no apparent enrichment of mTOR mRNA was detected (**Figure S5B**). This result suggested that miR-100-mediated regulation of mTOR expression is independent of Ago2. RISCs containing other Argonaute proteins (e.g., Ago1) may be utilized by miR-100 to recruit mTOR mRNA. Alternatively, miR-100 may regulate the expression of mTOR gene through a novel RISC-independent pathway. As a control, we also tested the enrichment of FOSL1 mRNA, a known miR-138 targeting gene without a miR-100 targeting site, in the RIP-IP assay. As shown in **Figure S5C**, a statistically significant enrichment of FOSL1 was observed in cells treated with miR-138, and no difference was detected in cells treated with miR-100. These results, together with the luciferase reporter assays, provided solid evidence supporting that members of miR-99 family down-regulate the expression of the AKT1 gene by directly interacting with AKT1 mRNA. As such, our results demonstrated that in addition to IGF1R and mTOR, ATK1 is another functional target gene of miR-99 family members in the AKT/mTOR signaling pathway.

Taken together, these results suggest that the miR-99 family plays an important role in dermal wound healing by concurrently targeting IGF1R, mTOR, and AKT1 (as well as AKT2, to a lesser extent), which in turn modulate the PI3K/AKT and mTOR signaling pathways. Our working hypothesis is that upon wounding, down-regulation of the miR-99 family members leads to the up-regulation/activation of AKT/mTOR signaling pathway, which in turn actives cell proliferation and migration, and facilitates the wound closure. In the late phase, miR-99 family members return to the basal levels, which will suppress the AKT/mTOR signaling and slow down cell proliferation and migration. The critical roles of the PI3K/AKT and mTOR pathways in wound healing have been well-documented by a number of in vitro and in vivo studies [38], [40]–[43]. It is worth noting that miR-99 family members have also been shown to regulate additional genes that participate in mTOR signaling, including Raptor [32], [44], an essential component of mTOR Complex 1 (mTORC1). While we confirmed the effect of the miR-99 family on Raptor in an oral cancer cell line (UM1), we did not observe any apparent change in Raptor expression after transfecting the HaCaT cells with miR-99a, miR-99b or miR-100 mimic (data not shown). This apparent difference in the effect of the miR-99 family on Raptor may be due to the differences in the cell lines tested. It is possible that the HaCaT cells (or the UM1 cells, or the adrenocortical tumor cell line and HeLa cells used in the previous studies [32], [44]) may have a specific mutation(s) that dictates the miR-99 family’s effects on Raptor. Alternatively, the previously observed effect of miR-99 family members on Raptor may be specific to malignancy. More in-depth functional analysis will be needed to fully evaluate the effect of the miR-99 family on Raptor and mTORC1. In addition to the PI3K/AKT and mTOR signaling pathways, the miR-99 family has also been implicated in other pathways, including regulating cell cycle by targeting proto-oncogene PLK1 [34], [45]–[47], an early trigger for G2/M transition. However, we were not able to observe any miR-99 family-mediated down-regulation of PLK1 in the cell lines we tested (data not shown). Nevertheless, our results, together with the earlier observations, suggest that miR-99 family members are multi-functional molecular regulators, and appear to play major roles in wound healing and other biological/pathological events.

In summary, we identified a panel of differentially expressed microRNAs during dermal wound healing, including members of the miR-99 family. We further demonstrated that miR-99 regulates cell migration and cell proliferation by targeting PI3K/AKT and mTOR signaling pathways during wound healing. Further studies are required to explore miR-99 family’s potential as a novel therapeutic target for the treatment of impaired wound healing.

## Supporting Information

Figure S1 **The expression of miR-99 family members in human skin wounds.** Skin wounds were created on healthy human subjects as described previously [Kiecolt-Glaser et. al.,: Slowing of wound healing by psychological stress. Lancet. 1995, 346(8984):1194-6], and the relative levels of miR-99a, miR-99b, and miR-100 on unwounded skin, and skin biopsy samples harvested at 6 hours and 1 day post-wounding (n = 3) were measured as described. This study was approved by Institutional Review Boards at University of Illinois at Chicago. *: p<0.05.
(PPT)

Figure S2 **The effect of anti-miR-100 treatment on cell proliferation and cell migration.** HaCaT cells were treated with anti-miR-100 LNA or negative control LNA. Cell proliferation was measured by MTT assay (**A**) and cell migration was measured by scratch assay (**B**). The experiments were performed in quadruplicates. *: p<0.05.
(PPT)

Figure S3 **The effect of miR-100 on IGF1R, mTOR, AKT1 mRNA levels in mouse skin wounds.** Experimental wounds were created as described. The wounds were treated with an ectopic application of 25 μL of miR-100 mimic (Dharmacon) or negative control (Dharmacon) with LipofectAMINE (Invitrogen) dissolved in saline solution at a final concentration of 2 μM. The wounds were covered with Tegaderm (3 M) (to avoid removal of the solution during grooming). Tissue samples were harvested at 1 day post-wounding/microRNA treatments. The relative levels of IGF1R, mTOR and AKT1 were measured by qRT-PCR as described (n = 6).
(PPT)

Figure S4 **Predicted miR-99 family targeting sites in AKT1 mRNA.** (**A**) Alignment of the sequences containing the predicted miR-99 family targeting sites in AKT1 mRNA among 11 species. Species key: hsa = human, ptr = chimpanzee, mml = rhesus, mmu = mouse, rno = rat, cfa = dog, fca = cat, eca = horse, bta = cow, ete = tenrec, oan = platypus. The miR-99 family targeting sites were identified by lines below the alignment. (**B**) The miR-100 targeting sites. (**C**) The miR-99a targeting sites. (**D**) The miR-99b targeting sites. The minimum free energy (mfe) for the binding of microRNAs to the targeting sequences were predicted using the RNAhybrid program [Krüger & Rehmsmeier: RNAhybrid: microRNA target prediction easy, fast and flexible. Nucleic Acids Res. 2006 Jul 1;34(Web Server issue):W451-4].
(PPT)

Figure S5 **MicroRNA-100-directed enrichment of IGF1R mRNA in the RISC complex.** RIP-IP assays were performed using a FLAG antibody as described in the Materials and Methods section to co-IP the Ago2 complexes from cells transfected with either miR-100 mimic or negative control mimic. qRT-PCR assays were performed on RNA samples isolated from the Ago2 co-IP fractions to measure the relative enrichment of the IGF1R and mTOR mRNA. An apparent enrichment of IGF1R was observed, but the change was not statistically significant (p = 0.11). No difference was observed in mTOR. As a control, we also tested the miR-138-mediated enrichment of FOSL1, a known miR-138 targeting gene [Jin et al.,: Molecular characterization of the microRNA-138-Fos-like antigen 1 (FOSL1) regulatory module in squamous cell carcinoma. J Biol Chem 2011, 286∶40104-9] with no known miR-100 targeting site, in the Ago2 co-IP fractions. An apparent enrichment of FOSL1 was observed in cells treated with miR-138, and no difference was observed in cells treated with miR-100. *: p<0.05.
(PPT)

Table S1 **Differential expression of microRNA in would healing.**
(DOC)

Table S2 **Common molecular pathways regulated by 63 differentially expressed microRNAs.**
(DOC)

Table S3 **Common molecular pathways regulated by 9 differentially expressed microRNAs.**
(DOC)
