# Supplementary figures and images for: MicroRNA-99 Family Targets AKT/mTOR Signaling Pathway in Dermal Wound Healing
Source: PLoS One. 2013 May 28;8(5):e64434. doi: 10.1371/journal.pone.0064434 (PMC3665798; doi:10.1371/journal.pone.0064434)

## Slide 1
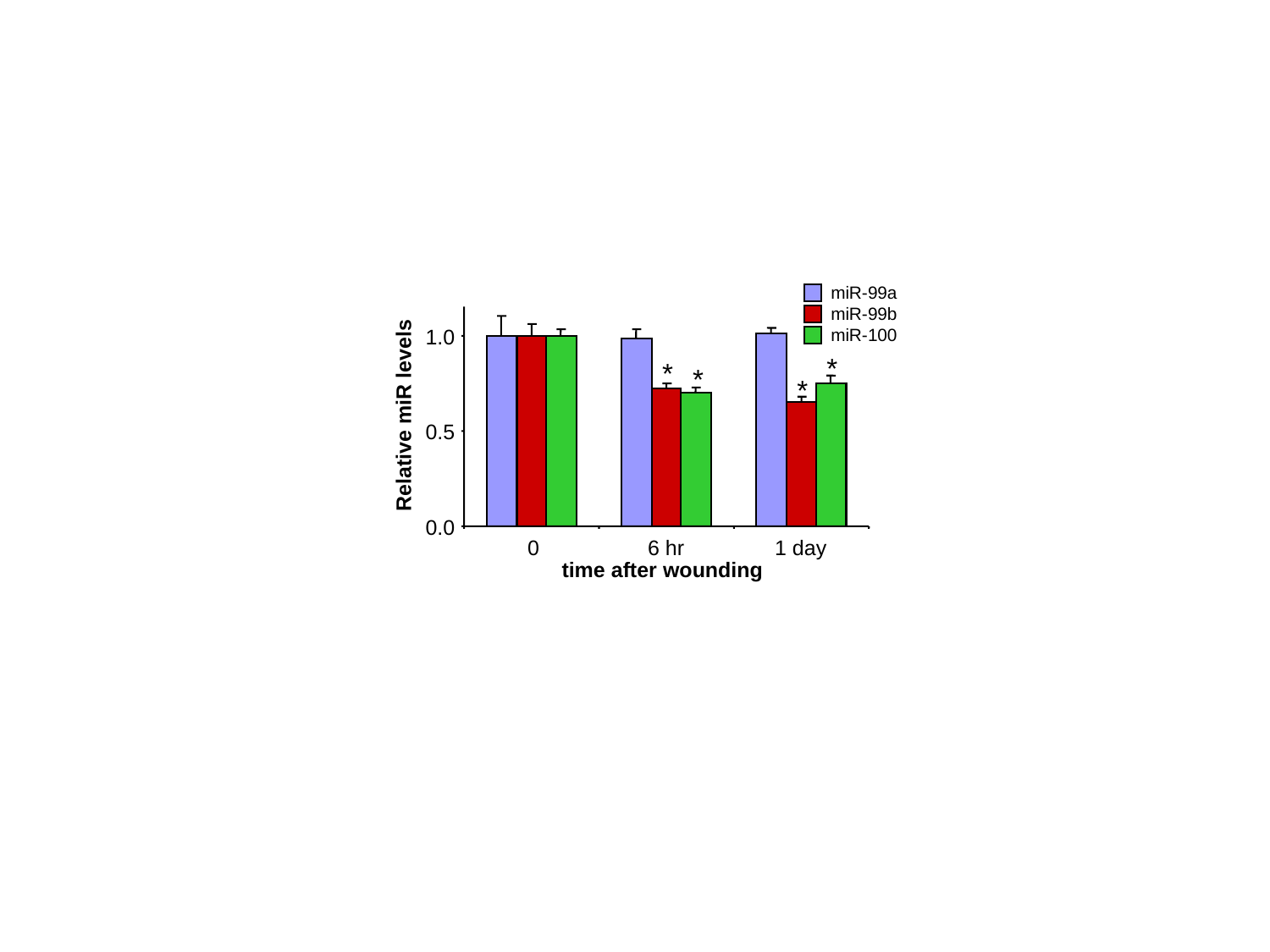

miR-99a
miR-99b
miR-100
1.0
*
*
*
*
Relative miR levels
0.5
0.0
0
6 hr
1 day
time after wounding

Supplement: Figure S1 — The expression of miR-99 family members in human skin wounds. Skin wounds were created on healthy human subjects as described previously [Kiecolt-Glaser et. al.,: Slowing of wound healing by psychological stress. Lancet. 1995, 346(8984):1194-6], and the relative levels of miR-99a, miR-99b, and miR-100 on unwounded skin, and skin biopsy samples harvested at 6 hours and 1 day post-wounding (n = 3) were measured as described. This study was approved by Institutional Review Boards at University of Illinois at Chicago. *: p<0.05. (PPT) [file pone.0064434.s001.ppt]

## Slide 1
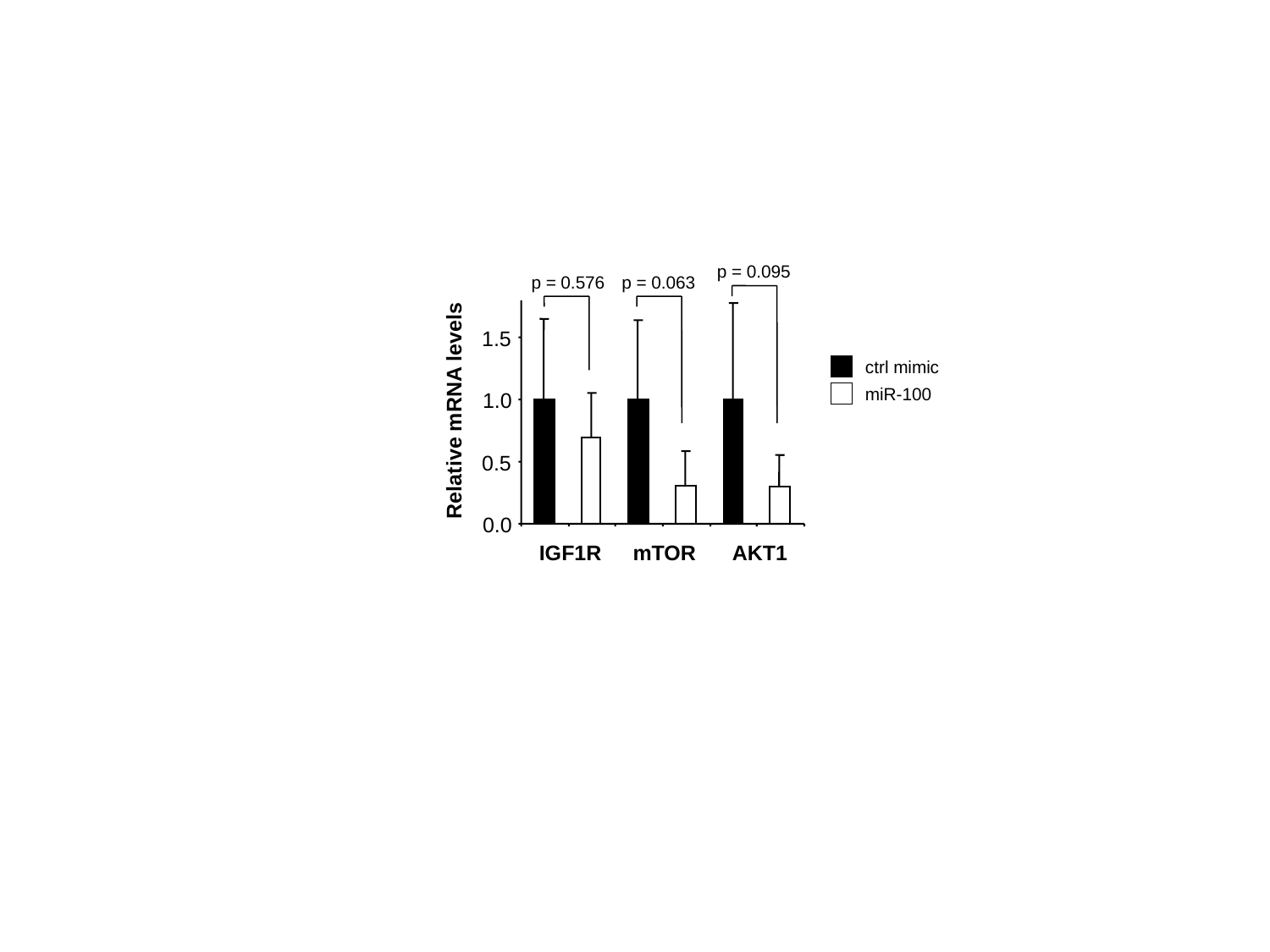

p = 0.095
p = 0.063
p = 0.576
 1.5
1.0
 0.5
0.0
ctrl mimic
miR-100
Relative mRNA levels
IGF1R
mTOR
AKT1

Supplement: Figure S3 — The effect of miR-100 on IGF1R, mTOR, AKT1 mRNA levels in mouse skin wounds. Experimental wounds were created as described. The wounds were treated with an ectopic application of 25 μL of miR-100 mimic (Dharmacon) or negative control (Dharmacon) with LipofectAMINE (Invitrogen) dissolved in saline solution at a final concentration of 2 μM. The wounds were covered with Tegaderm (3 M) (to avoid removal of the solution during grooming). Tissue samples were harvested at 1 day post-wounding/microRNA treatments. The relative levels of IGF1R, mTOR and AKT1 were measured by qRT-PCR as described (n = 6). (PPT) [file pone.0064434.s003.ppt]
