# Supplementary material for: MicroRNA-99 Family Targets AKT/mTOR Signaling Pathway in Dermal Wound Healing
Source: PLoS One. 2013 May 28;8(5):e64434. doi: 10.1371/journal.pone.0064434 (PMC3665798; doi:10.1371/journal.pone.0064434)
Supplement: Figure S2 — The effect of anti-miR-100 treatment on cell proliferation and cell migration. HaCaT cells were treated with anti-miR-100 LNA or negative control LNA. Cell proliferation was measured by MTT assay (A) and cell migration was measured by scratch assay (B). The experiments were performed in quadruplicates. *: p<0.05. (PPT) [file pone.0064434.s002.ppt]

## Slide 1
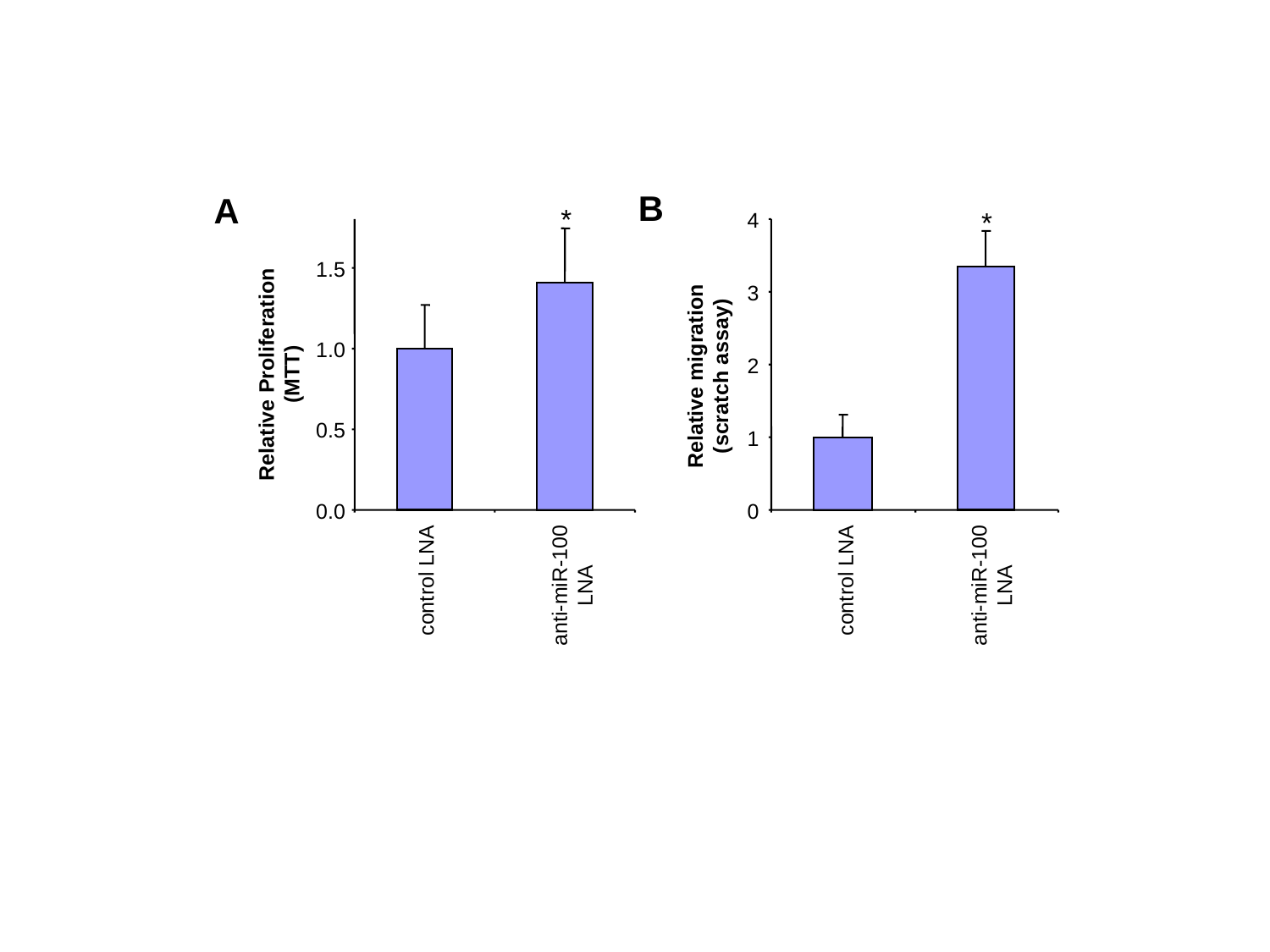

B
A
*
*
4
3
2
1
0
1.5
1.0
0.5
0.0
Relative Proliferation
(MTT)
Relative migration
(scratch assay)
anti-miR-100
LNA
anti-miR-100
LNA
control LNA
control LNA
1
0.5
0
