# Supplementary material for: MicroRNA-99 Family Targets AKT/mTOR Signaling Pathway in Dermal Wound Healing
Source: PLoS One. 2013 May 28;8(5):e64434. doi: 10.1371/journal.pone.0064434 (PMC3665798; doi:10.1371/journal.pone.0064434)
Supplement: Figure S4 — Predicted miR-99 family targeting sites in AKT1 mRNA. (A) Alignment of the sequences containing the predicted miR-99 family targeting sites in AKT1 mRNA among 11 species. Species key: hsa = human, ptr = chimpanzee, mml = rhesus, mmu = mouse, rno = rat, cfa = dog, fca = cat, eca = horse, bta = cow, ete = tenrec, oan = platypus. The miR-99 family targeting sites were identified by lines below the alignment. (B) The miR-100 targeting sites. (C) The miR-99a targeting sites. (D) The miR-99b targeting sites. The minimum free energy (mfe) for the binding of microRNAs to the targeting sequences were predicted using the RNAhybrid program [Krüger & Rehmsmeier: RNAhybrid: microRNA target prediction easy, fast and flexible. Nucleic Acids Res. 2006 Jul 1;34(Web Server issue):W451-4]. (PPT) [file pone.0064434.s004.ppt]

## Slide 1
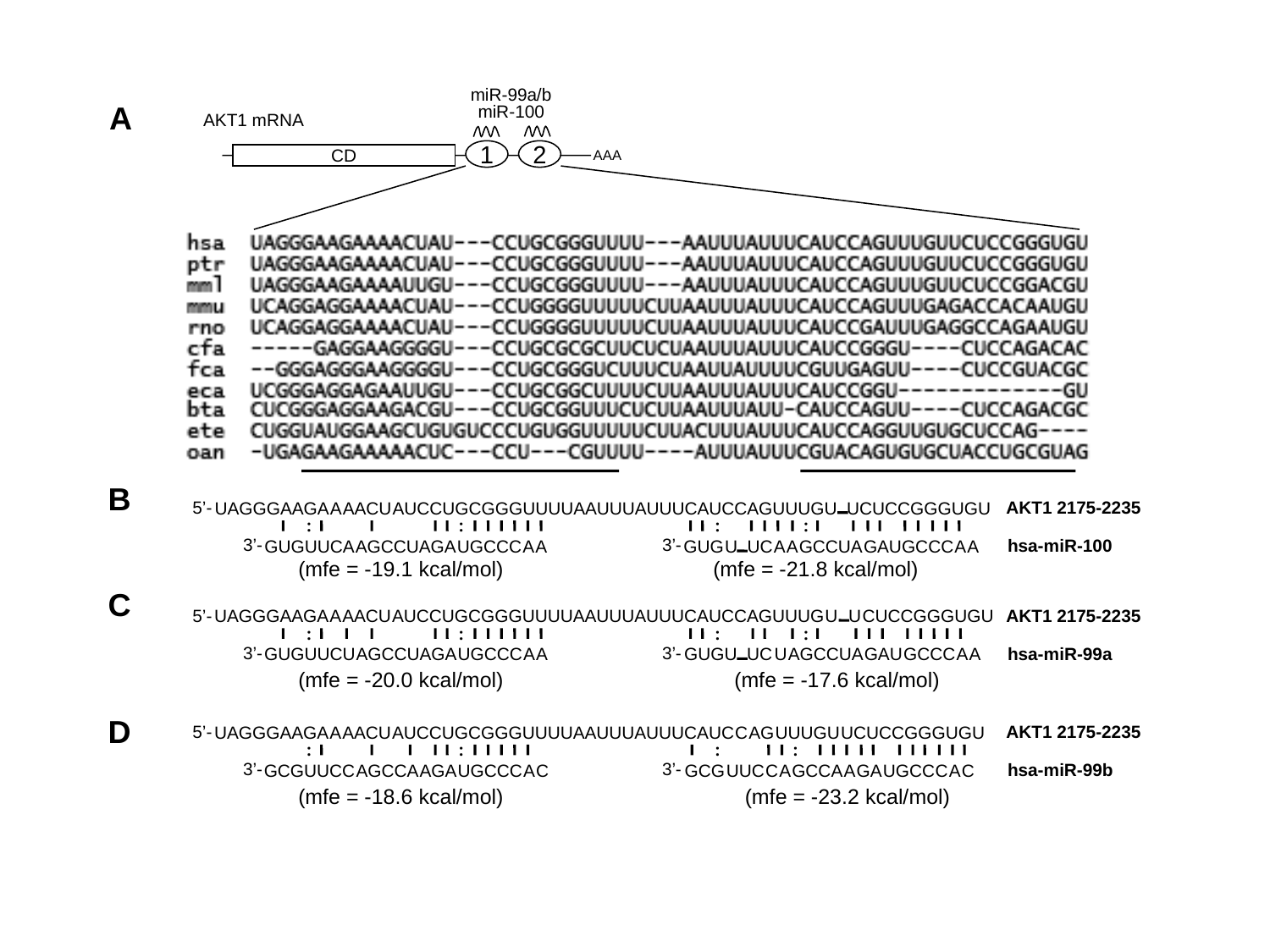

miR-99a/b
miR-100
AKT1 mRNA
AAA
1
2
CD
A
B
5’-
AKT1 2175-2235
UAGGGAAGA A AACU AUCCUGCGGGUUUUAAUUUAUUUCAUCCAGUUUGU UCUCCGGGUGU
 GUGUUCA AGCCUAGA UGCCC A A GUG U UC A A GCCUA GAUGCCC A A
:
:
:
:
3’-
3’-
hsa-miR-100
(mfe = -19.1 kcal/mol)
(mfe = -21.8 kcal/mol)
C
5’-
AKT1 2175-2235
UAGGGAAGA A AACU AUCCUGCGGGUUUUAAUUUAUUUCAUCCAGUUUG U U CUCCGGGUGU
 GUGUUCU AGCCUAGA UGCCC A A GUGU UC U AGCCUA GAU GCCC A A
:
:
:
:
3’-
3’-
hsa-miR-99a
(mfe = -20.0 kcal/mol)
(mfe = -17.6 kcal/mol)
D
5’-
AKT1 2175-2235
UAGGGAAGA A AACU AUCCUGCGGGUUUUAAUUUAUUUCAUC C AG UUUGU UCUCCGGGUGU
 GCGUUCC AGCCA AGA UGCCC A C GCG UUC C A GCCA A GA UGCC C A C
:
:
:
:
3’-
3’-
hsa-miR-99b
(mfe = -18.6 kcal/mol)
(mfe = -23.2 kcal/mol)
