# Supplementary material for: MicroRNA-99 Family Targets AKT/mTOR Signaling Pathway in Dermal Wound Healing
Source: PLoS One. 2013 May 28;8(5):e64434. doi: 10.1371/journal.pone.0064434 (PMC3665798; doi:10.1371/journal.pone.0064434)
Supplement: Figure S5 — MicroRNA-100-directed enrichment of IGF1R mRNA in the RISC complex. RIP-IP assays were performed using a FLAG antibody as described in the Materials and Methods section to co-IP the Ago2 complexes from cells transfected with either miR-100 mimic or negative control mimic. qRT-PCR assays were performed on RNA samples isolated from the Ago2 co-IP fractions to measure the relative enrichment of the IGF1R and mTOR mRNA. An apparent enrichment of IGF1R was observed, but the change was not statistically significant (p = 0.11). No difference was observed in mTOR. As a control, we also tested the miR-138-mediated enrichment of FOSL1, a known miR-138 targeting gene [Jin et al.,: Molecular characterization of the microRNA-138-Fos-like antigen 1 (FOSL1) regulatory module in squamous cell carcinoma. J Biol Chem 2011, 286∶40104-9] with no known miR-100 targeting site, in the Ago2 co-IP fractions. An apparent enrichment of FOSL1 was observed in cells treated with miR-138, and no difference was observed in cells treated with miR-100. *: p<0.05. (PPT) [file pone.0064434.s005.ppt]

## Slide 1
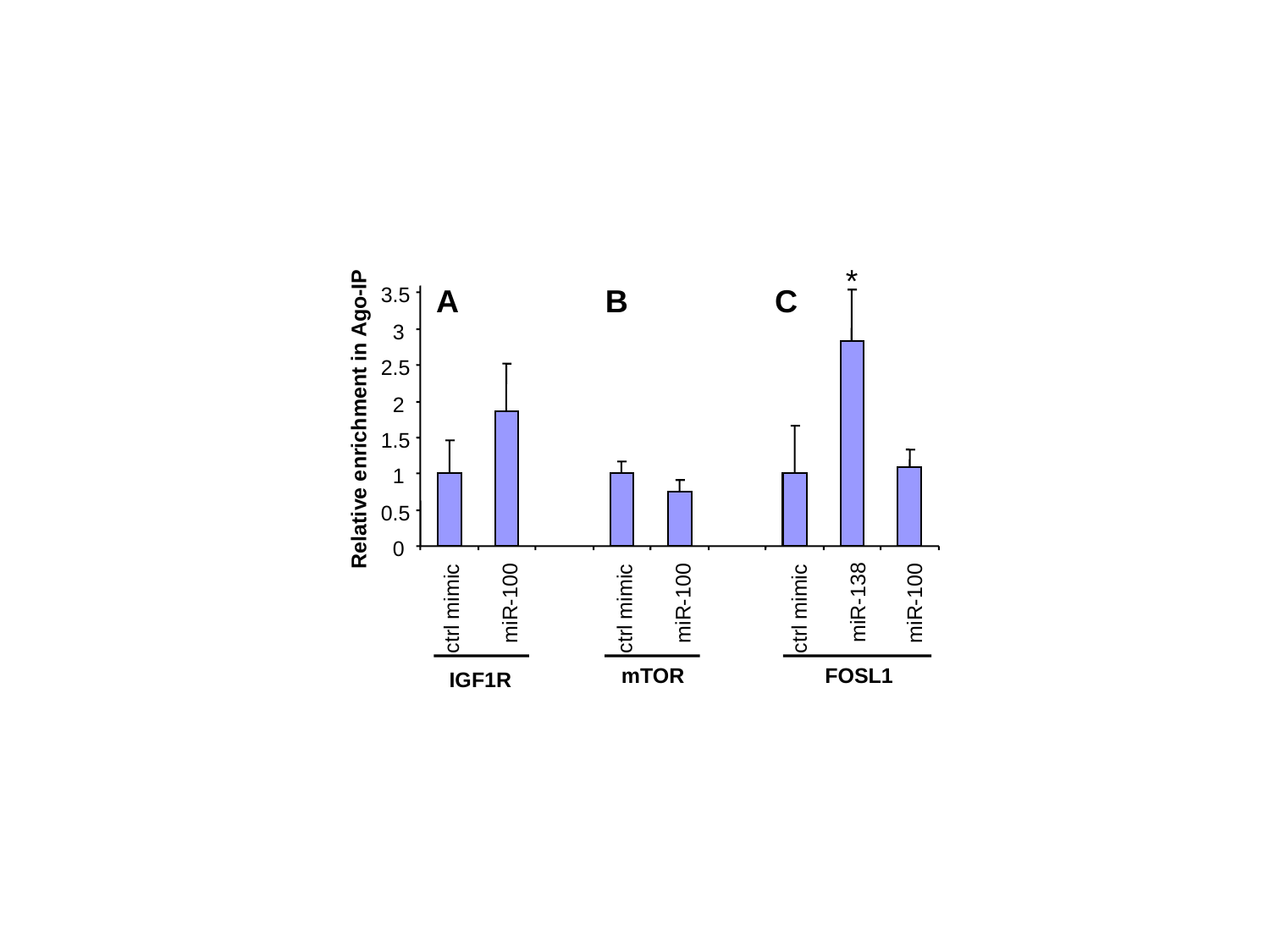

*
3.5
3
2.5
2
Relative enrichment in Ago-IP
1.5
1
0.5
0
miR-138
miR-100
miR-100
miR-100
ctrl mimic
ctrl mimic
ctrl mimic
mTOR
FOSL1
IGF1R
A
B
C
