# Supplementary material for: MicroRNA-99 Family Targets AKT/mTOR Signaling Pathway in Dermal Wound Healing
Source: PLoS One. 2013 May 28;8(5):e64434. doi: 10.1371/journal.pone.0064434 (PMC3665798; doi:10.1371/journal.pone.0064434)
Supplement: Table S1 — Differential expression of microRNA in would healing. (DOC) [file pone.0064434.s006.doc]

**Table S1: Differential expression of microRNA in would healing ^a^**

| **microRNA** | **Day 0** | **Day 1** | **Day 5** | **p-value** |
| --- | --- | --- | --- | --- |
| mmu-miR-182 | 1231 | 1069 | 400 | 0.000181 |
| mmu-miR-185 | 736 | 1015 | 314 | 0.00047 |
| mmu-miR-30d | 4945 | 4606 | 2920 | 0.000863 |
| mmu-miR-151-5p | 1973 | 2051 | 872 | 0.000879 |
| mmu-miR-423-5p | 1206 | 1846 | 504 | 0.000924 |
| mmu-miR-152 | 2553 | 1373 | 2643 | 0.00151 |
| mmu-miR-92a | 5472 | 5879 | 2036 | 0.00157 |
| mmu-miR-146a | 9888 | 6666 | 4315 | 0.00234 |
| mmu-miR-365 | 814 | 113 | 356 | 0.00296 |
| mmu-miR-92b | 2177 | 2529 | 768 | 0.00297 |
| mmu-miR-125a-5p | 7786 | 2471 | 4582 | 0.00436 |
| mmu-miR-714 | 113 | 223 | 514 | 0.00523 |
| mmu-miR-2137 | 988 | 7322 | 4824 | 0.00587 |
| mmu-miR-361 | 1644 | 2219 | 685 | 0.00593 |
| mmu-miR-1839-5p | 698 | 495 | 224 | 0.00609 |
| mmu-miR-199b* | 336 | 179 | 1730 | 0.0077 |
| mmu-miR-2141 | 297 | 667 | 918 | 0.00812 |
| mmu-miR-146b | 8845 | 9326 | 6025 | 0.0082 |
| mmu-miR-128 | 489 | 430 | 195 | 0.00846 |
| mmu-miR-99a | 2726 | 661 | 2487 | 0.0118 |
| mmu-miR-199a-3p | 17070 | 14411 | 20556 | 0.0121 |
| mmu-miR-1939 | 136 | 89 | 1339 | 0.0125 |
| mmu-miR-2146 | 654 | 2411 | 1825 | 0.0128 |
| mmu-miR-214 | 8491 | 7514 | 13300 | 0.0139 |
| mmu-miR-2145 | 526 | 716 | 1305 | 0.0149 |
| mmu-miR-199a-5p | 968 | 658 | 2862 | 0.015 |
| mmu-let-7d* | 483 | 221 | 335 | 0.0152 |
| mmu-miR-100 | 2158 | 389 | 1854 | 0.0154 |
| mmu-miR-2138 | 1860 | 4333 | 4911 | 0.0157 |
| mmu-miR-127 | 547 | 285 | 981 | 0.0159 |
| mmu-miR-132 | 272 | 584 | 198 | 0.0162 |
| mmu-miR-762 | 11024 | 23504 | 23808 | 0.0162 |
| mmu-miR-183 | 1443 | 970 | 261 | 0.0167 |
| mmu-miR-139-5p | 451 | 1434 | 397 | 0.0179 |
| mmu-miR-221 | 2507 | 1403 | 3154 | 0.018 |
| mmu-miR-1949 | 84 | 393 | 95 | 0.0187 |
| mmu-miR-151-3p | 484 | 358 | 228 | 0.0207 |
| mmu-miR-486 | 779 | 858 | 267 | 0.0209 |
| mmu-miR-2861 | 11920 | 22208 | 26672 | 0.0213 |
| mmu-miR-2134 | 1378 | 2502 | 4412 | 0.0216 |
| mmu-miR-689 | 2724 | 1608 | 8384 | 0.0216 |
| mmu-miR-19b | 336 | 184 | 1575 | 0.022 |
| mmu-miR-200b | 8370 | 4763 | 2535 | 0.0233 |
| mmu-miR-200c | 9976 | 8861 | 3538 | 0.0252 |
| mmu-miR-22 | 1317 | 803 | 3973 | 0.0262 |
| mmu-miR-2135 | 141 | 149 | 547 | 0.0268 |
| mmu-miR-223 | 5728 | 25289 | 10797 | 0.0271 |
| mmu-miR-181d | 580 | 174 | 373 | 0.0272 |
| mmu-miR-2133 | 505 | 2302 | 1403 | 0.0285 |
| mmu-miR-429 | 2446 | 1273 | 1133 | 0.0289 |
| mmu-miR-320 | 4612 | 4218 | 3238 | 0.0322 |
| mmu-miR-705 | 12061 | 21974 | 22454 | 0.0337 |
| mmu-miR-744 | 455 | 422 | 264 | 0.0355 |
| mmu-miR-690 | 3324 | 3298 | 5798 | 0.0362 |
| mmu-miR-10b | 7221 | 3929 | 2691 | 0.0386 |
| mmu-miR-25 | 4467 | 4917 | 3411 | 0.0407 |
| mmu-miR-30c | 7599 | 4135 | 6426 | 0.041 |
| mmu-miR-98 | 4901 | 486 | 1192 | 0.0423 |
| mmu-miR-125b-5p | 15908 | 8661 | 15596 | 0.0425 |
| mmu-miR-10a | 2576 | 866 | 841 | 0.0433 |
| mmu-miR-1894-3p | 2899 | 7381 | 10050 | 0.0449 |
| mmu-miR-652 | 436 | 467 | 301 | 0.0459 |
| mmu-miR-15b | 8041 | 11157 | 4744 | 0.0465 |

**^a^** For each time point, mean hybridization intensity was presented based on 3 independent microarray experiments on 3 animals.
