# Supplementary material for: MicroRNA-99 Family Targets AKT/mTOR Signaling Pathway in Dermal Wound Healing
Source: PLoS One. 2013 May 28;8(5):e64434. doi: 10.1371/journal.pone.0064434 (PMC3665798; doi:10.1371/journal.pone.0064434)
Supplement: Table S2 — Common molecular pathways regulated by 63 differentially expressed microRNAs. (DOC) [file pone.0064434.s007.doc]

**Table S2: Common molecular pathways regulated by 63 differentially expressed microRNAs^a^**

| **KEGG Pathway** | **Pathway ID** | **# of genes targeted in the pathway (Union)** | **-ln(p-value) ^b^** |
| --- | --- | --- | --- |
| Regulation of actin cytoskeleton | mmu04810 | 110 | 38.7 |
| MAPK signaling pathway | mmu04010 | 125 | 35.63 |
| Focal adhesion | mmu04510 | 100 | 34.2 |
| Axon guidance | mmu04360 | 74 | 33.69 |
| Glioma | mmu05214 | 43 | 29.1 |
| ErbB signaling pathway | mmu04012 | 52 | 26.29 |
| Melanoma | mmu05218 | 45 | 25.66 |
| Renal cell carcinoma | mmu05211 | 44 | 24.63 |
| Wnt signaling pathway | mmu04310 | 70 | 19.26 |
| mTOR signaling pathway | mmu04150 | 33 | 18.35 |
| Non-small cell lung cancer | mmu05223 | 33 | 18.35 |
| Prostate cancer | mmu05215 | 48 | 18.01 |
| Long-term potentiation | mmu04720 | 38 | 17.94 |
| Oxidative phosphorylation | mmu00190 | 6 | 16.35 |
| Colorectal cancer | mmu05210 | 45 | 16.08 |
| Chronic myeloid leukemia | mmu05220 | 41 | 15.55 |
| Insulin signaling pathway | mmu04910 | 64 | 15.12 |
| Pancreatic cancer | mmu05212 | 39 | 14.44 |
| Acute myeloid leukemia | mmu05221 | 33 | 14.02 |
| Adherens junction | mmu04520 | 39 | 13.89 |
| Endometrial cancer | mmu05213 | 30 | 13.25 |
| Ribosome | mmu03010 | 3 | 13.25 |
| Arachidonic acid metabolism | mmu00590 | 1 | 13.22 |
| Ubiquitin mediated proteolysis | mmu04120 | 59 | 13 |
| Metabolism of xenobiotics by cytochrome P450 | mmu00980 | 1 | 12.62 |
| GnRH signaling pathway | mmu04912 | 46 | 12.1 |
| TGF-beta signaling pathway | mmu04350 | 43 | 11.68 |
| Melanogenesis | mmu04916 | 46 | 11.29 |
| Gap junction | mmu04540 | 43 | 10.86 |
| T cell receptor signaling pathway | mmu04660 | 44 | 10.74 |
| Phosphatidylinositol signaling system | mmu04070 | 35 | 10.31 |
| Long-term depression | mmu04730 | 36 | 9.31 |
| Autoimmune thyroid disease | mmu05320 | 3 | 9.1 |
| Tight junction | mmu04530 | 54 | 8.25 |
| Type II diabetes mellitus | mmu04930 | 24 | 8.16 |
| Complement and coagulation cascades | mmu04610 | 6 | 7.38 |
| Fc epsilon RI signaling pathway | mmu04664 | 34 | 6.94 |
| Linoleic acid metabolism | mmu00591 | 2 | 6.94 |
| Histidine metabolism | mmu00340 | 1 | 6.57 |
| Taste transduction | mmu04742 | 5 | 6.53 |
| Small cell lung cancer | mmu05222 | 37 | 6.48 |
| Androgen and estrogen metabolism | mmu00150 | 2 | 6.34 |
| Glutathione metabolism | mmu00480 | 2 | 6.14 |
| Glycolysis / Gluconeogenesis | mmu00010 | 4 | 6.06 |
| Tryptophan metabolism | mmu00380 | 4 | 6.06 |
| Antigen processing and presentation | mmu04612 | 10 | 6.03 |
| Pyrimidine metabolism | mmu00240 | 11 | 5.88 |
| Bile acid biosynthesis | mmu00120 | 1 | 5.75 |
| Allograft rejection | mmu05330 | 4 | 5.68 |
| Dorso-ventral axis formation | mmu04320 | 14 | 5.33 |
| Fatty acid metabolism | mmu00071 | 3 | 5.21 |
| Heparan sulfate biosynthesis | mmu00534 | 11 | 5.07 |
| Valine, leucine and isoleucine degradation | mmu00280 | 3 | 5.02 |

**^a^** Of the 63 differentially expressed microRNAs, 50 were present in the DIANA-mirPath database, and were used for the analysis. 13 microRNAs were not present in the database, including miR-125b-5p, miR-128, miR-1839-5p, miR-1894-3p, miR-199b*, miR-2133, miR-2134, miR-2135, miR-2137, miR-2138, miR-2141, miR-2145, miR-2146.

**^b^** Computed using DIANA-mirPath [Papadopoulos et al.,: DIANA-mirPath: Integrating human and mouse microRNAs in pathways. Bioinformatics 2009, 25:1991-3].
