# Supplementary material for: MicroRNA-99 Family Targets AKT/mTOR Signaling Pathway in Dermal Wound Healing
Source: PLoS One. 2013 May 28;8(5):e64434. doi: 10.1371/journal.pone.0064434 (PMC3665798; doi:10.1371/journal.pone.0064434)
Supplement: Table S3 — Common molecular pathways regulated by 9 differentially expressed microRNAs. (DOC) [file pone.0064434.s008.doc]

**Table S3: Common molecular pathways regulated by 9 differentially expressed microRNAs^a^**

| **KEGG Pathway** | **Pathway ID** | **# of genes targeted in the pathway (Union)** | **-ln(p-value) ^b^** |
| --- | --- | --- | --- |
| Long-term potentiation | mmu04720 | 24 | 21.62 |
| mTOR signaling pathway | mmu04150 | 19 | 16.35 |
| MAPK signaling pathway | mmu04010 | 56 | 15.76 |
| Axon guidance | mmu04360 | 34 | 15.71 |
| Glioma | mmu05214 | 21 | 15.7 |
| Ubiquitin mediated proteolysis | mmu04120 | 34 | 15.15 |
| Regulation of actin cytoskeleton | mmu04810 | 47 | 14.11 |
| ErbB signaling pathway | mmu04012 | 24 | 12.37 |
| Prostate cancer | mmu05215 | 24 | 11.45 |
| Melanogenesis | mmu04916 | 25 | 10.44 |
| Acute myeloid leukemia | mmu05221 | 17 | 9.57 |
| Insulin signaling pathway | mmu04910 | 31 | 9.38 |
| GnRH signaling pathway | mmu04912 | 23 | 8.26 |
| Colorectal cancer | mmu05210 | 21 | 8.2 |
| Renal cell carcinoma | mmu05211 | 18 | 7.82 |
| T cell receptor signaling pathway | mmu04660 | 22 | 7.46 |
| TGF-beta signaling pathway | mmu04350 | 21 | 7.3 |
| O-Glycan biosynthesis | mmu00512 | 9 | 7.03 |
| Focal adhesion | mmu04510 | 37 | 6.96 |
| Oxidative phosphorylation | mmu00190 | 2 | 6.86 |
| Phosphatidylinositol signaling system | mmu04070 | 17 | 6.24 |
| Inositol phosphate metabolism | mmu00562 | 13 | 5.94 |
| Type II diabetes mellitus | mmu04930 | 12 | 5.29 |
| Endometrial cancer | mmu05213 | 13 | 5.18 |
| Non-small cell lung cancer | mmu05223 | 13 | 5.18 |
| Fc epsilon RI signaling pathway | mmu04664 | 17 | 5.03 |

**^a^** 9 differentially expressed microRNAs (in cluster X from **Figure 1A**, including miR-152, miR-365, let-7d*, miR-125a-5p, miR-181d, miR-99a, miR-100, miR-30c, miR-125b-5p) were used for the analysis. Pathway analysis with a complete list of differentially expressed microRNAs is presented in **Supplementary Table 2**.

**^b^** Computed using DIANA-mirPath [Papadopoulos et al.,: DIANA-mirPath: Integrating human and mouse microRNAs in pathways. Bioinformatics 2009, 25:1991-3].
